# Os ACOD Apresentam o Melhor Custo-Benefício na Prevenção da Fibrilação Atrial na Vida Real?

**DOI:** 10.36660/abc.20200120

**Published:** 2020-04-06

**Authors:** Márcio Bittencourt

**Affiliations:** Universidade de São PauloHospital Universitário de São PauloDivisão de Medicina InternaSão PauloSPBrasilUniversidade de São Paulo - Hospital Universitário de São Paulo - Divisão de Medicina Interna, São Paulo, SP - Brasil

**Keywords:** Acidente Vascular Cerebral (AVC)/prevenção e controle, Fibrilação Atrial, Anticoagulantes/administração e dosagem, Varfarina/economia, Análise de Custo Benefício

O Sr. D., um professor universitário aposentado de 75 anos de idade com história de um acidente vascular cerebral (AVC) prévio, acorda de manhã, dirige até o hospital para coletar sangue e ajustar sua dose de varfarina e depois vai para o seu trabalho. Algumas horas depois, ele recebe uma ligação da equipe de enfermagem, informando como ajustar a dose: “A partir de hoje, o senhor deve tomar 7,5 mg de varfarina às segundas, quartas e sextas-feiras. Nos outros dias da semana, o senhor pode manter o comprimido de 5 mg a que está acostumado. Se o senhor não encontrar o comprimido de 7,5 mg, pode cortar o de 5 mg ao meio e tomar um comprimido e meio nesses dias. Não é muito complicado, né? A propósito, lembre-se de não abusar de couve e espinafre que eu sei que o senhor gosta!”. Não fosse o fato de o Sr. D. também tomar enalapril e atenolol para a hipertensão arterial e controlar a frequência cardíaca de sua fibrilação atrial (FA), uma estatina para prevenção secundária desde o AVC e metformina para o diabetes; cortar comprimidos ao meio e lembrar em que dia ele deve tomar qual dose não deveria ser muito complicado.

O Sr. D. é o paciente comum com FA não-valvar atendido em clínicas de consultórios particulares no Brasil e em outros lugares do mundo, embora os pacientes do Sistema Único de Saúde (SUS) usualmente passem muito mais tempo no hospital aguardando os resultados pessoalmente ou retornando no dia seguinte para verificá-los devido aos recursos mais limitados para contatar os pacientes por telefone.

Como a maioria dos pacientes em uso de varfarina enfrenta essas complexidades, não surpreende que seu uso adequado na vida real esteja longe do ideal.^1^ Em média, os pacientes passam pelo menos um terço do tempo acima ou abaixo da razão normalizada internacional (INR, *international normalized ratio*)^2^ e, apenas um em cada quatro pacientes apresente uma INR terapêutica estável por 6 meses consecutivos. E mesmo entre esses, apenas um terço permanece com uma INR terapêutica estável no ano seguinte, de acordo com dados dos Estados Unidos (EUA).^1^Infelizmente, no Brasil, América Latina e outros países com status socioeconômico mais baixo, o tempo dentro da faixa terapêutica (TTR, *time within the therapeutic range*) para a INR é menor do que o relatado para os EUA ou a Europa, mesmo em ensaios randomizados.^3^ Nesses países, outros desafios da vida real para obter anti-coagulação adequada levaram à sua subutilização, incluindo acesso limitado às medidas de INR em áreas rurais e outras restrições de recursos.^4^ Além disso, dados recentes sugerem que aproximadamente um quarto de todo o custo relacionado ao uso de varfarina está relacionado ao tempo de viagem e aos custos associados às medidas de INR na Finlândia, e esses custos geralmente não são cobertos por nenhuma empresa de seguro de saúde.^5^

Dentro do contexto de tais restrições no uso de varfarina, o desenvolvimento de anticoagulantes orais diretos (ACOD), onde não é necessário monitoramento, e uma dose fixa pode ser utilizada, foi ansiosamente esperado pela comunidade médica. Esses medicamentos não apenas demonstraram ser mais eficazes e mais seguros do que a varfarina em um estudo randomizado de pacientes com FA não valvar,^6^ mas resultados comparáveis foram observados em um grande registro nos EUA.^7^ Além disso, os ACOD provavelmente têm bom custo-benefício no Reino Unido.^8^ No entanto, a realidade de prática clinica na vida real, bem como as implicações de custo, são altamente variáveis e podem não ser facilmente reproduzidos em outros países. Por exemplo, os ACOD atualmente não são cobertos pelo SUS no Brasil. Assim, são necessários dados sobre resultados e estudos de custo-efetividade com foco na reprodução desses estudos em outros cenários, como no Brasil.

O artigo de Barros e Silva,^9^ et al., publicado na edição atual, fornece dados brasileiros de pacientes com FA não-valvar que recebem anti-coagulação oral com cobertura por um provedor de seguro privado.^9^ Seus resultados sugerem que, pelo menos para aqueles cobertos por um grande plano de seguro de saúde privado, os padrões e implicações do uso de varfarina versus ACOD no Brasil se assemelham aos padrões de outros países. Primeiro, apenas cerca de metade do INR estava dentro da faixa terapêutica e, em média, os pacientes passaram quase metade do tempo fora do alvo terapêutico, como relatado anteriormente. Mais importante, ao passar menos de 65% do tempo dentro da faixa terapêutica foi associado a um aumento de três vezes no risco de sangramento maior, de 1,6% para 5,3%. Por fim, os custos diretos associados a esses eventos devastadores foram substanciais, mais de R$ 25.000 por membro ao ano. Embora nenhuma análise formal de custo-efetividade tenha sido realizada no presente estudo, os resultados parecem estar alinhados com o recente estudo do Reino Unido, e os ACOD provavelmente apresentarão melhor custo-benefício se os eventos hemorrágicos adversos forem mais baixos do que as taxas encontradas com o uso de varfarina, pois esses eventos são caros. Além disso, com o ônus associado ao monitoramento da INR, é mais provável que o uso de ACOD seja custo-efetivo na perspectiva da sociedade. Coletivamente, o presente estudo apoia a ideia geral de que o ACOD deveria ser com cobertura de seguro privado no Brasil. No entanto, devido às diferenças significativas nos padrões de custos e práticas entre os sistemas de saúde público e privado no Brasil, são necessários dados mais robustos do SUS antes da incorporação dos achados atuais em rotinas da prática clínica no sistema público de saúde, mesmo que as expectativas sejam de que pacientes como o Sr. D. estariam melhor sem a necessidade de comparecer à avaliação mensal da INR.
