# Supplementary material for: Os ACOD Apresentam o Melhor Custo-Benefício na Prevenção da Fibrilação Atrial na Vida Real?
Source: Arq Bras Cardiol. 2020 Apr 6;114(3):467–8. [Article in Portuguese] doi: 10.36660/abc.20200120 (PMC7792714; doi:10.36660/abc.20200120)
Supplement: Translation

# Translation: Are DOACs a Good Bang for Your Buck in Atrial Fibrillation Prevention in Real-Life?

**Authors:** Márcio Bittencourt

**Affiliations:** BrazilUniversidade de São Paulo - Hospital Universitário de São Paulo - Divisão de Medicina Interna, São Paulo, SP - Brazil

**Keywords:** Stroke/prevention and control, Atrial Fibrillation, Anticoagulants/administration and dosage, Varfarin/economy, Cost-Benefit Analysis

Mr. D., a 75-year-old retired university professor with a prior stroke, wakes up in the morning, drives to the hospital to have blood drawn to adjust his warfarin dosage and then goes to work. A couple of hours later, he gets a call from the nursing team telling him how to adjust the dose: “starting today you should take 7.5 mg of warfarin on Mondays, Wednesdays and Fridays. On the other days of the week you can keep up with the 5 mg pill you are used to. If you do not have a 7.5 mg pill you can cut the 5 mg in half and take one and a half pills on those days. It is not too complicated, is it? By the way, remember to go slow on that kale and spinach I know you like!”. Were it not for the fact the Mr. D. also takes enalapril and atenolol for his blood pressure and to control the heart rate of his atrial fibrillation (AF), a statin for secondary prevention since the stroke, and metformin for his diabetes; cutting pills in half and remembering on what day he should take which dosage should be too complicated.

Unfortunately, Mr. D. is about the average non-valvular AF patient seen in clinics in private practices in Brazil and places around the world, though patients from the Brazilian Unified Health System (*Sistema Unico de Saúde*, SUS) usually spend substantially more time at the hospital waiting for the results in person or coming back the next day to check them, due to more limited resources to contact patients over the phone.

Since most patients using warfarin face such complexities, it should come as no surprise that its real life adequate use is far from ideal.^1^ Patients on average spend at least a third of their time above or below target international normalized ratio (INR) values.^2^ Interestingly, only about one in every four patients has a stable therapeutic INR during 6 consecutive months. And even among those, only a third remains with a stable therapeutic INR over the following year, according to data from the United States (U.S.).^1^ Unfortunately, in Brazil, Latin America and other countries with lower socioeconomic status, the time within the therapeutic range (TTR) for INR is shorter than those reported for the U.S. or Europe even in randomized trials.^3^ In such countries, other real-life challenges to attain adequate anticoagulation has led to its underuse, including limited access to INR measurements in rural areas, and other constraints in resources.^4^ Moreover, recent data suggests that approximately one quarter of the entire cost related to warfarin use is related to the travel time and costs associated with INR measurements in Finland, and such costs are usually not covered by any health insurance company.^5^

Within the context of such constraints in warfarin use, the development of direct oral anticoagulants (DOAC), where no monitoring is needed, and a fixed dose can be used is highly expected by the medical community. Not only did these drugs have shown to be more effective and safer than warfarin in a randomized trial of non-valvular AF patients,^6^but comparable results were seen in a large U.S. registry.^7^Moreover, DOACs are likely to be cost effective in the United Kingdom.^8^ However, the real-life practice patterns, as well as cost implications, are highly variable and might not be easily reproduced in other countries. For example, DOACs are not currently covered by the SUS in Brazil. Thus, data on outcomes and cost-effectiveness studies focusing on the reproduction of such studies in other scenarios, such as in Brazil, are needed.

The manuscript by Barros e Silva, et al.,^9^ published in the current issue, provides Brazilian data from patients with non-valvular AF receiving oral anticoagulation and covered by a private insurance provider.^9^ Their results suggest that, at least for those covered by a large private healthcare insurance plan, the patterns and implications of warfarin vs. DOAC use in Brazil resembles the patterns in other countries. First, only about half of the INR were within the therapeutic range, and on average patients spent almost half of their time outside the target, as reported previously. More importantly, spending less than 65% of the time within the therapeutic range was associated with a three-fold increase in the risk of major bleeding, from 1.6% to 5.3%. Finally, the direct costs associated with such devastating events was substantial, more than R$25,000 per member per year. Although no formal cost-effectiveness analysis was performed in the present study, the findings seem to be in line with the recent UK study, and DOAC are likely to be more cost effective if adverse bleeding events are lower, as such events are costly. Additionally, with the burden associated with INR monitoring, the use of DOACs is even more likely to be cost-efficient from a societal perspective. Collectively, the present study supports the overall idea that DOAC should be the preferred choice with private insurance coverage in Brazil. However, due to the significant differences in cost and practice patterns between private and public healthcare systems in Brazil, more robust SUS data is needed prior to the translation of current findings into routine practice in the public health care system, even if the expectations are that patients like Mr. D. would be better off without their the need to come for their monthly INR assessment.
